# Treatment of FANCA Cells with Resveratrol and N-Acetylcysteine: A Comparative Study

**DOI:** 10.1371/journal.pone.0104857

**Published:** 2014-08-15

**Authors:** Marta Columbaro, Silvia Ravera, Cristina Capanni, Isabella Panfoli, Paola Cuccarolo, Giorgia Stroppiana, Paolo Degan, Enrico Cappelli

**Affiliations:** 1 SC Laboratory of Musculoskeletal Cell Biology, IOR, Bologna, Italy; 2 DIFAR-Biochemistry Lab., University of Genova, Genova, Italy; 3 CNR-National Research Council of Italy, Institute of Molecular Genetics, Unit of Bologna-IOR, Bologna, Italy; 4 S. C. Mutagenesis, IRCCS AOU San Martino – IST (Istituto Nazionale per la Ricerca sul Cancro), Genova, Italy; 5 Centro di Diagnostica Genetica e Biochimica delle Malattie Metaboliche, Istituto Giannina Gaslini, Genova, Italy; 6 Hematology Unit, Istituto Giannina Gaslini, Genova, Italy; University of Padova, Italy

## Abstract

Fanconi anemia (FA) is a genetic disorder characterised by chromosome instability, cytokine ipersensibility, bone marrow failure and abnormal haematopoiesis associated with acute myelogenous leukemia. Recent reports are contributing to characterize the peculiar FA metabolism. Central to these considerations appears that cells from complementation group A (FANCA) display an altered red-ox metabolism. Consequently the possibility to improve FA phenotypical conditions with antioxidants is considered. We have characterized from the structural and biochemical point of view the response of FANCA lymphocytes to N-acetyl-cysteine (NAC) and resveratrol (RV). Surprisingly both NAC and RV failed to revert all the characteristic of FA phenotype and moreover their effects are not super imposable. Our data suggest that we must be aware of the biological effects coming from antioxidant treatment.

## Introduction

Fanconi anemia (FA) is a genetic disorder characterised by chromosome instability and cytokine hypersensitivity. Bone marrow failure and abnormal haematopoiesis are associated with high frequency to clonal hematopoietic stem cells (HSC) expansion, acute myelogeneous leukemia via a mechanism involving genomic instability and inflammation [1].

We recently reported [2], [3] different structural abnormalities in FANCA cells underlying an impaired mitochondrial functionality affecting the energy metabolism. A defective respiration at the mitochondrial oxidative phosphorylation complex I is associated with a reduced ATP production and altered ATP/AMP ratio. These defects are consistently associated with impaired oxygen metabolism [4]. Therefore the possibility to improve FA patients physiological state with antioxidants as therapy adjuvants appears promising.

N-acetyl-cysteine (NAC) is a sulphydryl-group providing compound which acts as a precursor of reduced glutathione (GSH) and as direct scavenger of reactive oxygen species (ROS). Intracellular reduced GSH is often depleted as a consequence of increased oxidative stress and inflammation. Hence NAC can regulate the red-ox status in the cells interfering with several signalling pathways. NAC is widely used in clinical treatment [5] as support in treatment of diseases related to oxidative stress. Actually almost 250 studies with NAC are enlisted in the Clinical Trials governmental registry (www.clinicaltrials.gov).

Resveratrol (RV) is a naturally-occurring polyphenol mainly found in grapes. A growing body of literature has demonstrated the beneficial effects of RV on age-related metabolic deterioration and its protective role in metabolic diseases. RV exerts its potent anticarcinogenic effect by inducing apoptosis and inhibiting tumor promoter-induced cell transformation [6]. RV protects against the deregulation of energy homeostasis, up-regulates eNOS and many cellular anti-oxidant enzymes, down regulates TNFα and NF-κB expression and inhibits NADPH oxidases [7]. Moreover, crystallographic studies showed that RV (and other related polyphenols) directly inhibits the rotary mechanism of mitochondrial F_o_F_1_-ATP synthase (ATP synthase) by binding to a site in γ-subunit and hence its ATP synthetic activity [8]. More than 70 clinical trials with RV are actually listed in the National Institutes of Health website.

In FA, the use of NAC and RV has already been proposed. Treatment with NAC, in association with Lipoic Acid (LA) [9] increased cellular viability as well as GSH and ATP contents, and reduced spontaneous and DEB-induced chromosomal instability in lymphocyte from FA patients. The protective abilities of NAC, RV and tempol were compared in the FANCD2 murine model [10]. NAC and RV partially corrected the abnormal cell cycle state of the HSP cells and helped maintaining them in a quiescent state. In turn tempol substantially delayed tumor onset apparently without a beneficial effect on hematopoiesis. Finally an antioxidant dietary formulate containing lysine, proline, ascorbic acid and green tea extracts, was successfully reported to inhibit in vitro and in vivo FANCA-associated head and neck squamous cell carcinoma [11].

Notwithstanding the potential interest concerning these results the still crucial and open question is that we do not know yet which molecular mechanisms and metabolic pathways are relevant in the FA pathological phenotype. Here we evaluate the biological effects of RV and NAC, two most promising antioxidants which act with different biochemical mechanisms.

## Materials & Methods

### Ethics statement

Study approval was obtained from the Ethics Committee at the Gaslini Hospital, Genova, Italy (protocol N° J5002 date: 24/9/2010). Informed written consent was obtained from the adult subjects and from parents, on the behalf of their children, involved in the study. All clinical investigations were conducted according to the principles expressed in the Declaration of Helsinki.

### Cell culture and treatments

FANCA primary fibroblast cell lines, isogenic FANCA primary fibroblasts corrected with S11FAIN [12] retrovirus and wild type (wt) cells were grown as monolayer at 37°C in RPMI supplemented with 10% fetal calf serum and antibiotics. All the cell lines between the 5th and 15th passage, were grown with the same density and conditions. During treatment we didn’t observe significant changes in the cellular viability. FANCA and wt lymphoblast cell lines were grown at 37°C in RPMI supplemented with 10% fetal calf serum and antibiotics. Primary lymphocytes were isolated using Ficoll-Paque Plus and grown at 37°C in RPMI supplemented with 10% fetal calf serum, antibiotics and phytohemagglutinin (20 µg/ml). N-acetyl-cysteine (500 µM), resveratrol (10 µM) were added to the cells once a day for five days. One hour after the last treatment, cells were used for protein extracts or fixed for electron microscopy experiments. FANCA corrected cells always behaved alike wt [3].

### Oxygen consumption measurements

To measure oxygen consumption, an amperometric electrode (Unisense-Microrespiration, Unisense A/S, Denmark) was used. Experiments were performed in a closed chamber at 25°C. For each experiment 500.000 cells were permeabilized with 0.03 mg/ml digitonin for 1 minute, centrifuged for 9 minutes at 1000 rpm and resuspended in: 137 mM NaCl, 0.7 mM NaH_2_PO_4_, 5 mM KCl and 25 mM Tris HCl pH 7.4. The same medium was used in the oximetric experiments. To stimulate the pathway composed by the respiratory complexes I, III and IV, 10 mM pyruvate and 5 mM malate was added to the sample, while to stimulate the pathway II, III and IV 20 mM succinate was used as respiratory substrate. To confirm that the oxygen consumption was really due to oxidative phosphorylation (OXPHOS) machinery, 0.1 mM rotenone or 0.2 mM antimycin A were used as inhibitors for the first and second pathway, respectively [13].

### Electron transfer from Complex I to Complex III

The electron transfer between Complex I to Complex III was studied spectrophotometrically, following the reduction of cytochrome c at 550 nm. The extinction molar coefficient used for reduced cytochrome c was 1 mM^−1^ cm^−1^. For each assay, 50 µg of total proteins was used. The assay medium containing; 100 mM Tris-HCl pH 7,4 and 0,03% cytochrome c. The reaction was started with the addition of 0,7 mM NADH [3]. If the electron transport between Complex I and Complex III is conserved, the electrons pass from NADH to Complex I, then to Complex III via coenzyme Q, and finally to cytochrome c. 

### Adenylate kinase (AK) assay

AK activity was assayed spectrophotometrically, following NADH oxidation at 340 nm [14]. ATP and GTP in the assay mixture are needed to assay the ATP-AMP phosphotransferase activity (AK1 + AK2) or the GTP-AMP phosphotransferase activity (mitochondrial AK3), respectively. Results are reported for the activity of the enzyme (U/mg) per sample.

### ATP and AMP quantification

ATP and AMP were measured according to the enzyme coupling method of Bergmeyer et al. [15]. For ATP assay, medium contained 20 µg of sample, 50 mM Tris- HCl pH 8.0, 1 mM NADP, 10 mM MgCl2, and 5 mM glucose in 1 ml final volume. Samples were analyzed spectrophotometrically before and after the addition of 4 µg of purified hexokinase/glucose-6-phosphate dehydrogenase (Boehringer). The rise in absorbance at 340 nm, due to NADPH formation, was proportional to the ATP concentration. For AMP assay, the medium contained 20 µg of sample, 50 mM Tris-HCl pH 8.0, 1 mM NADH, 10 mM MgCl2, and 10 mM phosphoenolpyruvate (PEP), 2 mM ATP in 1 ml final volume. Samples were analyzed spectrophotometrically before and after the addition of 4 µg of purified pyruvate kinase/lactate dehydrogenase (Boehringer). The rise in absorbance at 340 nm, due to NADH oxidation, was proportional to the AMP concentration. For all biochemical experiments, protein concentrations were determined using the Bradford method.

### Electron microscopy

Lymphocyte pellets from healthy donor and FANCA patients (subjected or not to antioxidant treatments) were fixed with 2.5% glutaraldehyde 0.1 M cacodylate buffer pH 7.6 for 1 h at room temperature. After post-fixation with 1% OsO4 in cacodylate buffer for 1 h, pellets were dehydrated in an ethanol series and embedded in Epon resin. Ultrathin sections stained with uranyl-acetate and lead citrate were observed with a Jeol Jem-1011 transmission electron microscope. Two hundred mitochondria were examined for each sample.

### Cytometry

ROS were quantified by cytometry with 2′,7′-dichlorodihydrofluorescein diacetate (H2DCFH-DA). This dye is a cell-permeable, nonfluorescent molecule, which is very sensitive to intracellular redox change. When H_2_DCFH-DA enters into the cell it is cleaved by intracellular esterases into 2′,7′-dichlorodihydrofluorescein (H_2_DCF) which, in presence of H_2_O_2_, oxidize to the fluorescent molecule dichlorofluorescein (DCF). Fluorescence from this probe is measured by flow cytometry, as the dye is excited by the 488-nm laser. H_2_DCFH-DA has a good specificity for H_2_O_2_, and it has been shown that the fluorescence of the product DCF appears to be mediated mainly by H_2_O_2_. Cytometry measures were performed on a Cyan ADP cytometer (Beckman Coulter, Mountain View, CA, USA) equipped with three laser lamps.Ten thousand cells per sample were analyzed, and the results are reported as the percentage of cells, relative to the relevant control, that display a fluorescence shift.

### Statistical analysis

Data were analyzed by one-way ANOVA and unpaired two-tail Student's t test using instat software (GraphPad Software, Inc., La Jolla, CA, USA). Data are expressed as mean ± standard deviation (SD) from 3 to 5 independent determinations performed in duplicate. In the figures SD are shown as error bars. An error probability with P<0.05 was selected as significant.

## Results

### 1 – Cytometric ROS quantification

An enhanced oxidative stress appears the common denominator of the many altered functions in FANCA cells. In fact, we reported [2], [3] an enhanced ROS production in FANCA cells associated with a scarce NADH utilization or availability at the mitochondrial respiratory Complex I. NAC and RV, although with different mechanisms as discussed below, provide significant reduction in intracellular ROS production. When FANCA cells were treated with NAC or RV a significant decrease (p<0,01) in intracellular ROS production was measured (Table 1). The large differences reported among the samples analysed might be attributed to the different functional characteristics of the mutant FANCA proteins produced. In this context, the structure-function relationships in mutant FANCA proteins (in the various patients) are however as yet not fully available to-date and cannot be properly discussed at this time. Castella et al. [19] reported cytoplasmic expression of mutated FANCA protein without functional correlation between genotype and phenotype. Moreover, this work evaluated the effect of FANCA mutated protein only in the perspective of the DNA repair activity without considering other possible functions for FANCA protein. Indeed, these putative functions may be the cause of the high variability observed.

**Table 1:** Cytometric quantification of the ROS reduction (%) in FANCA cells after treatment with NAC and RV.

Samples	ROS reduction (%)	
	+ NAC	+ RV
FANCA 1	32.67	31.28
FANCA 2	42.11	30.37
FANCA 3	27.70	37.03
FANCA 4	41.24	60.94
FANCA 5	41.40	28.71
FANCA 6	46.33	47.10
FANCA 7	35.85	64.18
FANCA 8	39.67	44.29
FANCA 9	44.81	57.17
FANCA 10	16.59	46.84
FANCA 11	59.26	n.d.
FANCA 12	45.50	43.94
FANCA 13	26.65	36.58
FANCA 14	77.84	16.31

### 2 – Biochemical Parameters

#### 2.1 - Oxygen consumption

**Figure 1:**
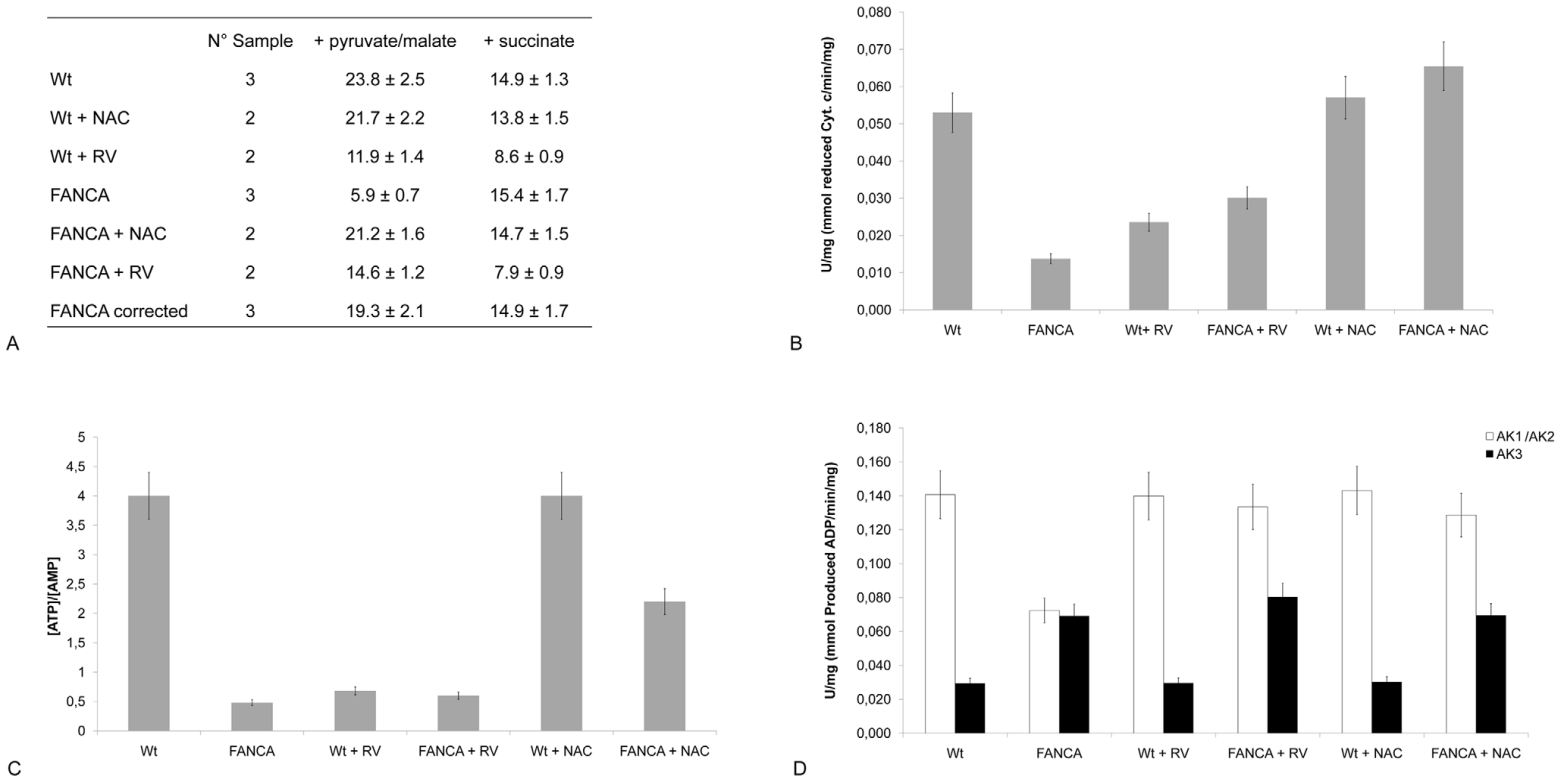
Biochemical parameters in FANCA and wild-type (wt) cells untreated or treated with 500 µm nac or 10 µm rv for 5 days. A. Table reports values for oxygen consumption (µM O_2_/min/mg) in FANCA and wild type (wt) primary fibroblasts treated NAC or RV after induction with pyruvate/malate (Complexes I, III and IV), or succinate (Complexes II+III+IV). B. Assay of the electron transfer from Complex I to Complex III on wt and FANCA samples. Electron transfer between Complex I and III was measured following the reduction of cytochrome c at 550 nm after the addition of 0.7 mM NADH, the substrate of Complex I. Data are reported as µmol reduced Cytochrome c/min/mg. C. Figure shows the ratio among ATP and AMP concentration in wt and FANCA samples. D. Histogram reports a comparison of the ATP-AMP phosphotransferase activity (AK1+AK2 white columns) and GTP-AMP phosphotransferase (AK3, black columns) activity on wt and FANCA samples. The activity is expressed as µmol of ADP produced/min/mg. Data are expressed as mean ± SD. Each panel is representative of at least five experiments.

As described by Ravera et al. [3], FANCA cells display defective respiration trough the pathway composed by Complex I, III and IV. In our experimental setting, FANCA and wild type (wt) primary fibroblasts were treated with 500 µM NAC or 10 µM RV for 5 days. In these conditions the ability of FANCA cells to respire from the Complex I, III and IV pathway were fully restored (Fig. 1A) after NAC and only partially after RV treatment.

Moreover RV reduced the ability of wt cells to respire both in the presence of pyruvate/malate and with succinate, as respiring substrates. This effect may be explained considering that RV is an inhibitor of the activity of ATP synthase [16], [17]. Namely, being oxygen consumption coupled to ATP synthesis, when ATP synthase is inhibited, the electron transport chain activity also slows down, and so does oxygen consumption. In conclusion, even though the effects of NAC and RV follow different pathways, the net results is a reduction of ROS generated at the level of Complex I. Genetic complementation by FANCA gene corrects the respiratory defect. The same results were obtained in lymphoblast cell lines from the same patients (data not shown).

#### 2.2 - Complex I to Complex III electron transfer

Defective respiration in FANCA cells is due to defect in electron transfer from Complex I to Complex III and is associated with reduced ATP production and altered ATP/AMP ratio [3]. However, as shown above, the treatment of FANCA cells with NAC restores their ability to respire through Complex I and, accordingly, electron transfer efficiency between Complex I and III is restored in FANCA at level comparable to wt cells. By contrast, RV only partially recovered the FANCA cells electron transfer efficiency. Also, in line with the results for oxygen consumption, it reduced the electron transfer ability in wt cells (Fig. 1B). The difference among the effect of NAC and RV likely depends on their different target. In fact, being a precursor of GSH NAC is really an antioxidant molecule able to decrease the damage due to ROS production consequent to impaired functioning of Complex I. By contrast, RV acts by inhibiting the activity of ATP synthase, thus reducing the speed of electron transport chain, i.e. of Complex I. This causes a lower production of ROS thus allowing cell scavengers systems to work.

#### 2.3 - Intracellular ATP and AMP quantification

In FANCA the ATP/AMP ratio is very low with respect to the control (Fig. 1C) in agreement with what can be expected from the impairment of Complexes I-III electron transport. By contrast, after NAC treatment, the ATP level in FANCA cells was restored and the AMP concentration decreases, although it remained higher than in wt, allowing to reach a good ATP/AMP ratio (Fig. 1C). In the case of RV treatment only a partial recovery of ATP was seen in FANCA and a strong reduction in wt cells was observed. This is likely related to the inhibitor effect of RV on ATP synthase, considering that ATP synthase is the principal source of ATP production for the cell.

#### 2.4 - Adenylate Kinase (AK) activity

Adenylate Kinase (AK) activities were also investigated. These enzymes catalyse the interconversion of adenine nucleotides and contributing to the regulation of the cellular energy homeostasis. In particular, we assayed the ATP-AMP phosphotransferase isoforms (AK1 and AK2), which catalyze the interconversion of ADP to ATP+AMP and the GTP-AMP phosphotransferase isoform, typical of the mitochondrial matrix, which utilizes GTP as donor for the phosphate group to AMP [3]. In FANCA cells ATP-AMP phosphotransferase activity was impaired while GTP-AMP phosphotransferase activity was increased (Fig. 1D). Both NAC and RV treatment restored FANCA ATP-AMP phosphotransferase activity, to a level similar to wt cells. By contrast, as already observed [3], the GTP-AMP phosphotransferase activity was higher in FANCA cells with respect to controls. Thus FANCA cells appear to preferably use GTP, from Krebs cycle, as alternative source of energy. This could be explained considering that in FANCA cells the electron transport among Complex I and III is impaired. Consequently ATP synthesis from oxidative phosphorylation is lower with respect to control. Interestingly, RV and NAC treatments do not restore the GTP-AMP phosphotransferase activity to the control level, suggesting that energetic metabolism restoration by the antioxidant treatment in FANCA cells is not complete.

### 3 – Electron Microscopy

**Figure 2:**
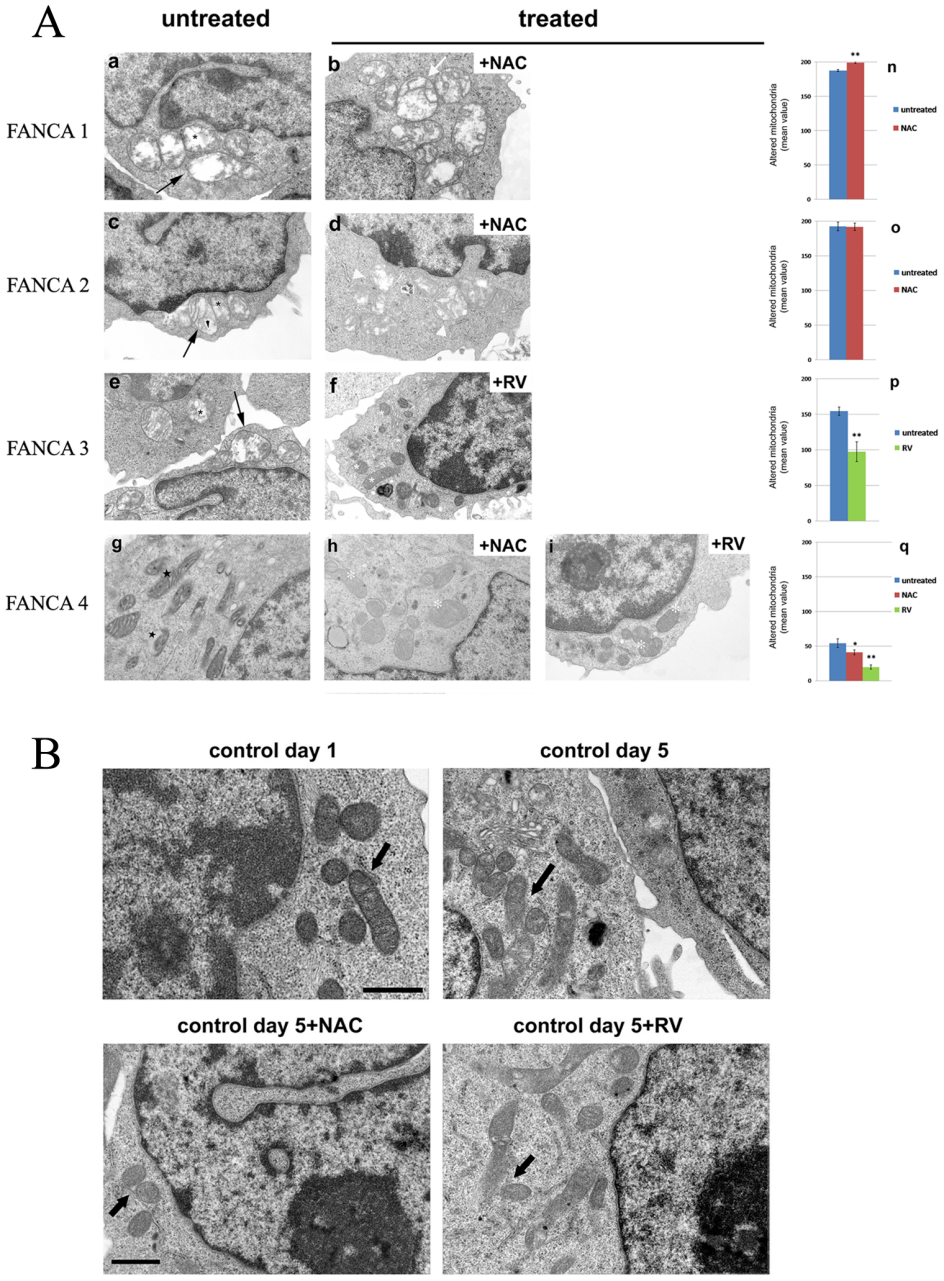
Morphological changes of mitochondria in FANCA lymphocytes and healthy controls. **A.** Representative transmission electron microscopy images of morphologically altered mitochondria from untreated (a,c,e,g) and treated FANCA (b,d,f,h,i) with different antioxidants. Mitochondria of untreated lymphocytes from FANCA patients 1, 2, 3 (a,c,e) appeared swollen (black arrows) with increased size, disrupted cristae (black asterisks) and rarefacted matrix (black arrowheads). On the contrary, mitochondria in untreated lymphocytes from FANCA4 patient were small, oval and round with dark matrix and swollen internal cristae (black stars). (b) Mitochondria of FA1 lymphocytes showed worsened alteration such as more enlarged shape (white arrow) after NAC treatment. (d) Mitochondria of FANCA2 lymphocytes appeared indistinguishable from untreated (b) in overall aspect, after NAC treatment (white arrowheads). (f) Mitochondria of FANCA3 and FANCA4 lymphocytes showed a striking rescue after NAC or RV treatments (white asterisks). (Scale bars, 1 µm) **B.** Representative micrographs of morphologically normal mitochondria (arrowheads) in control lymphocytes at day 1 and day 5 before and after NAC or RV treatments. (Scale bars 1µm).

By ultrastructural analysis (Fig. 2A), we observed striking mitochondrial changes in FANCA lymphocytes after NAC, RV or NAC plus RV treatments compared to untreated FANCA lymphocytes Indeed in NAC treated FANCA1 lymphocytes many mitochondria (30%) were greatly enlarged with disrupted cristae and rarefaction of matrix density (Fig. 2b) rather than the swollen shape mitochondrial observed in untreated FANCA1 lymphocytes (Fig. 2a). On the contrary, NAC treatment did not cause detectable changes in mitochondrial structure in FANCA2 lymphocytes (Fig. 2d) compared to untreated FANCA2 cells (Fig. 2c). In fact, before and after NAC treatment, most of the mitochondria appeared swollen with matrix rarefaction and altered cristae. Surprisingly, in RV treated FANCA3 lymphocytes a significant recovery in the mitochondria organization (40%) consisting in elongated, small or rod –like shape mitochondria with dense matrix and normal distribution of cristae (Fig. 2f), was seen compared to untreated FANCA3 lymphocytes characterized by altered swollen mitochondria with fewer cristae (80%) (Fig. 2e). In untreated FANCA4 lymphocytes most mitochondria had normal morphology, but 30% of them showed highly condensed matrix with swollen internal cristae (Fig. 2g), which are indicative of mitoptosis, a kind of dead mitochondria [18]. After NAC treatment the number of mitochondria in mitoptosis decreases and the cells shown normal mitochondrial structure (Fig. 2h). However, the number of apoptotic cells increased with about 30% of cells in apoptosis (data not shown). After RV treatments, we observed an increased of percentage of ameliorated mitochondria (20%) with dense matrix and regularly distributed cristae (Fig. 2i). Small, electron-dense granules were occasionally seen in untreated and treated FANCA4 lymphocytes.

Fig. 2B shows representative micrographs of morphologically normal mitochondria from control lymphocytes at day 1 and day 5 before and after NAC or RV treatments.

## Discussion

FANCA cells display an altered red-ox metabolism [4]. The possibility to improve this altered physiological conditions with the employment of antioxidants has been consequently forwarded/proposed. Here we evaluated the biological effects of two antioxidants featuring different biochemical mechanisms. While from the point of view of the biochemistry and the energy metabolism NAC treatment appears advantageous, the here reported results concerning cellular morphology suggests attention especially in the view to employ NAC as therapeutic in FA. On the contrary RV induces only a partial recovery of the biochemical parameters, but great improvement in cellular morphology.

Our data suggest that we must be aware of possible pitfalls of the employment of antioxidants as therapeutics. While short time exposure (from few hours to 2 days) to NAC appears beneficial in FA [9], [20], upon mere observation of the biochemical parameters, the effect of NAC appears positive for the FANCA cells. However when we extend our observation to the cellular ultrastructure, some cellular defects don’t improve and negative effects are seen, such as worsening of mitochondrial structure. A possible explanation for this could paradoxically be the recovery of electron transport chain functioning by NAC. The pro-oxidant FA phenotype cells is characterized by alterations in the electron transport efficiency in the inner mitochondrial membrane with loss of efficiency of electron transfer from Complex I to Complex III which can in fact be restored by administration of exogenous Coenzyme Q [3]. NAC decreases ROS production and normalizes mitochondrial respiration. Therefore it is expected that in FA cells with increased ROS production, oxygen consumption will increase in the presence of NAC (Fig. 1A). The ability of NAC to restore Complex I activity is however inherently linked to an increase in ROS production and therefore in worsening of the already existent structural damage typical of FACA cells. This is also suggestive of the existence of a primary damage to mitochondrial membranes in the FANCA phenotype. The sole increase in GSH reductive potential in FANCA cells appears insufficient to restore a fully functional phenotype. NAC was already reported [21] to be only partially effective in limiting GSH depletion, particularly when employed in drugs targeted to mitochondria.While the negative phenotypic outcomes from the chronic NAC treatment in FANCA cells were almost unexpected its possible detrimental effects are already known. The forced increase in intracellular GSH was indeed reported to induce a paradoxical triggering of mitochondrial oxidative stress [22]. After either pharmacologic (NAC supplementation) or genetic (glutamate cysteine ligase overexpression) maneuvers an initially more reducing GSH redox potential or reductive stress was promptly followed by a pathogenic mitochondrial oxidation. The GSH-mediated reductive stress culminated with pro-oxidative consequences in mitochondria contrary to the common belief that NAC functions solely as an antioxidant.

The various lymphocytes here analysed do show various types of mitochondrial alterations. It has been reported that no apparent correlation exists between the genotypic defect and the phenotypic expression [19]. However it also holds true that no molecular function or metabolic regulation other than DNA repair has been exploited to date.

The different response to NAC observed in the analysed samples may be correlated to the different genetic background. It appears clear, however, that the effect of NAC on the overall cell population examined is null or negative. In FANCA1 NAC treatment (fig. 2b) result in mitochondria with greatly enlarged, disrupted cristae and rarefaction in matrix density where in FANCA2 (fig. 2d) no detectable changes are seen in mitochondrial structure; in FANCA4 (fig. 2h) we observe a normal mitochondrial structure and a decrease in the number of mitochondria in mitoptoses but with a concomitant increase in the number of apoptotic cells. It appears peculiar that the level of the apoptotic cells in the NAC treated sample equals the level of mitoptotic cells in the untreated. It has been reported that ROS produced by the respiratory chain inside mitochondria could also induce mitochondrial fission as the initial step of mitoptosis [23]. The same degeneration in the mitochondrial reticulum here reported has been described by us in FA fibroblasts [2].

RV treatment partially restored the FANCA biochemical activity but resulted in significant recovery of a normal structure. It may appear surprising that in the presence of RV mitochondrial functions like ATP production and oxygen uptake are increased in FANCA cells (and decreased in wild type cells, as expected). To explain this apparent contradiction the cited defective phenotype of Complex I in FANCA cells [3], as well as the results from Gledhill et al. [8] should be taken into consideration. These Authors, by structural analysis of ATPase crystallized in the presence of RV demonstrated that it binds the inside surface of F_1 n_preventing both the synthetic and hydrolytic activities of ATPase [8]. In this respect, RV cannot be considered a mere ROS scavenger, rather its action is exerted also through mild control of the ATP synthase activity. Being an inhibitor of ATP synthase [8], RV slows down the electron transport chain activity and NADH utilization by Complex I which has the result to lower free radical production. It can be hypothesized that in these conditions aerobic respiration is more efficient and more directed to ATP production increasing O_2_ utilization finalized to H_2_O and not to ROS production. RV alone inhibits ATP synthase and decreases electron transport chain activity producing less free radicals. In fact, when the ATP synthase is inhibited also the electron transport chain activity slows, and consequently the oxygen consumption decreases with a kind of indirect scavenging.

RV was shown to reduce mitochondrial respiration, and Complex IV activity and to extend lifespan. In fact diminished mitochondrial OXPHOS is associated with lower ROS production and reduced longevity. A beneficial effect of RV in tumors may derive in part by its preventing mitochondrial ATP synthesis, thereby inducing apoptosis. RV was shown to decrease tumor cell viability, an effect that can be reverted by overexpression of Bcl-2 [24]. However, RV may exert other effects, also in dependence of the model used. For example, treatment of the whole animal (mice) *in vivo* with RV causes induction of OXPHOS genes, an effect likely related to activation of the protein deacetylase, SIRT1 [25].

We have also tested a combination of NAC and RV. Unfortunately, the double treatment did not restore the correct OXPHOS activity in FANCA-cells, nor the electron transport from Complex I to Complex III (data not shown). We may explain these results considering the single contribution from each compound: NAC restores the functionality of Complex I, while RV reduces mitochondrial respiration, and ATP synthesis, by reversibly inhibiting ATP synthase. In the FANCA case, the combination of the two has the effect of both blocking mitochondrial respiration and improving the functioning of Complex I: this would cause a backlog of electrons, augmenting ROS production and increasing oxidative damage to the mitochondrial inner membranes. Literature reports that a mild uncoupling between electron transport chain and ATP synthase induces a H^+^ leak and increases ROS production.

Ultrastructural analysis also showed that the combined treatment of NAC plus RV did not improve mitochondrial alterations (data not shown). Instead, concomitant treatment with RV and NAC worsened the mitochondrial phenotype (data not shown). In this case ATP synthase blockade by RV on one hand and promotion of electron transfer by NAC on the other produce an increase in oxidative damage.

In conclusion, both NAC and RV failed to revert the biochemical and structural phenotype FANCA phenotype. Moreover, their effects are not super imposable. Even though the NAC treatment did restore the OXPHOS functionality in FA cells, the phenotype did not improve; by contrast RV exerted a better effect on FA cell phenotype, although it was not able to revert all the characteristic of the mutant cells. Therefore, an antioxidant therapy is likely to be beneficial in several pathological conditions; however an evaluation of the effects of the single antioxidant employed should be carefully scrutinized to avoid possible detrimental consequences. Our knowledge of the proper use and the effects of any single antioxidant, and for their combined use as well, is as yet quite limited.
